# Functions of Egg-Coating Substances Secreted by Female Accessory Glands in Alderflies, Fishflies and Dobsonflies (Megaloptera)

**DOI:** 10.3390/insects13090766

**Published:** 2022-08-25

**Authors:** Pei Yu, Xingyue Liu, Fumio Hayashi

**Affiliations:** 1 Department of Biology, Tokyo Metropolitan University, Minamiosawa 1-1, Hachioji 192-0397, Tokyo, Japan; 2 Department of Entomology, China Agricultural University, Beijing 100193, China

**Keywords:** accessory gland material, Corydalidae, defensive secretion, egg predation, egg protection, Sialidae

## Abstract

**Simple Summary:**

Egg masses of the insect order Megaloptera are coated with female accessory gland substances and are diverse in shape, color, and surface structure. Female alderflies (family Sialidae) and fishflies (family Corydalidae, subfamily Chauliodinae) lay eggs in a single layer on the substrate (although the eggs are occasionally multi-layered in the fishfly genus *Nigronia*), and the accessory gland substances are usually brown. In contrast, dobsonflies (Corydalidae, Corydalinae) lay a multi-layered hemispherical egg mass, and the accessory gland substances differ in color and chemical properties among genera and species. Egg masses of the dobsonfly genus *Protohermes* are coated with a sticky substance, while those of other dobsonflies are covered with hardened white powders, both of which contribute to the prevention of egg desiccation. The accessory gland substances of most species of Megaloptera also function to protect eggs from attack by oophagous predators, such as ladybird beetles and ants. The information on the parasitoids of Megaloptera egg masses are also discussed based on the present and previously reported observations.

**Abstract:**

Eggs of insects are immobile and must endure harsh environmental conditions (e.g., low temperatures in winter and aridity in summer) and avoid attack by egg-eating predators, egg parasites, and microbes. Females of Megaloptera lay their eggs as a single- or multi-layered egg mass, which is coated with chemical substances secreted from the female reproductive accessory glands. In this study, we observed the egg masses laid by females of two species of Sialidae (alderflies), nine species of Chauliodinae (fishflies), and 23 species of Corydalinae (dobsonflies) belonging to the order Megaloptera and examined the functions of accessory gland substances coating the laid eggs. The female accessory gland is a single tube in alderflies and fishflies but a paired pouch in dobsonflies. The amount and color of the gland substances differ greatly among species. These substances prevent egg desiccation, inhibit egg feeding by ladybird beetles, and repel ants. Most characteristics of the egg mass structures and the effectiveness of accessory gland substances reflect the phylogeny of Megaloptera, although some differ among closely related taxa.

## 1. Introduction

Insect eggs are protected physically and chemically, to allow them to survive harsh environmental conditions and biological attacks. The chorion differs interspecifically in structure, reflecting different physiological needs and environmental hazards [1]. Deposited eggs are highly vulnerable to oophagous predators and parasitoids, and eggs are therefore also protected directly by the parents or indirectly by maternal secretory chemicals that cover the eggs [2,3]. Further, maternal secretion of chemical substances onto the laid eggs and the cleaning of each egg contributes to antimicrobial activity [4,5,6]. Most insects coat their eggs with substances produced in the female accessory glands, which is assumed to protect the eggs against predators, parasites, microorganisms, and desiccation [3,7].

Despite the significance of such diverse physical and chemical means of insect egg protection, little information is available for several insect groups [7,8]. The order Megaloptera, consisting of alderflies, fishflies, and dobsonflies, is one such understudied group. The shapes of egg masses have been described in a few species of alderflies (*Sialis*, *Protosialis*, *Ilyobius*), fishflies (*Neohermes*, *Chauliodes*, *Nigronia*), and dobsonflies (*Corydalus*, *Chloronia*, *Platyneuromus*) [9,10,11,12,13,14,15,16,17,18,19]. The egg mass in some species is associated with substances secreted from the abdominal tip of the female. The female alderfly *Sialis* seems to secrete a sticky substance from its abdomen onto the substrate before oviposition [20]. In the female dobsonfly *Corydalus*, the secreted substance may have adhesive properties that assist egg attachment to the substrate and to other eggs in the mass [13]. The female *Corydalus* secretes a fluid from the female abdomen that is spread over the laid egg mass [13]. After drying, this fluid changes to a hard white covering of the entire egg mass, which may protect the eggs from excessive heating because its white color reflects heat [13,21]. It is generally thought that female secretion protects eggs from predation and parasitism, but this has not been examined in Megaloptera.

In this study, we observe the shapes of egg masses and the substances to coat the egg masses by the females of two species of alderflies, nine species of fishflies, and 23 species of dobsonflies and examine the three putative functions of female accessory gland substances, i.e., protection of eggs from desiccation, repulsion of general predators, and avoidance of predation by oophagous predators. Finally, the effects of the shape of Megaloptera egg masses on parasitism by the egg parasitoids are discussed based on the data obtained in this study and the previous reports.

## 2. Materials and Methods

### 2.1. Examined Species

We observed the egg masses laid by females of two species of alderflies (Sialidae: *Sialis*), nine species of fishflies (Corydalidae: Chauliodinae; *Anachauliodes*, *Nigronia*, *Parachauliodes*, *Neochauliodes*), and 23 species of dobsonflies (Corydalidae: Corydalinae; *Protohermes*, *Nevromus*, *Neoneuromus*, *Acanthacorydalis*) (Table 1). Light-trapped females in the field and those emerging from laboratory-reared larvae were placed in individual glass vessels (65 mm in diameter, 90 mm in height) at 25 ± 1 °C (14:10 h L:D cycle) after measuring their head width (between the outer margin of the right and left eyes) and forewing length (distance from the tip to the base) using slide calipers. A wet filter paper was placed on the bottom of the vessel to prevent desiccation, and the top was covered with nylon mesh and a glass lid. Adults were given 10% sucrose solution every day, which was dropped onto the adult mouthpart until they stopped drinking.

Field-collected larvae were placed in individual glass vessels (65 mm in diameter, 90 mm in height) with small stones on the bottom acting as refuges. Well-aerated tap water, not exceeding 5 mm in depth, was provided and replaced daily. The larvae were fed one or two living last-instar larvae of a chironomid midge every day. The rearing vessels were stored in an incubator at a constant temperature of 15 ± 1 °C, 20 ± 1 °C, or 30 ± 1 °C (14:10 h L:D cycle) according to the water temperature of the larval habitat of each species. When the larvae stopped feeding for one week, they were individually relocated to moist peat moss, in which they made holes for pupation, and maintained at 25 ± 1 °C (14:10 h L:D cycle). Adults usually emerged after about a 14-day prepupal period and about a 10-day pupal period.

The two egg masses parasitized by the egg parasitoids were found in the field and preserved in 80% ethanol. The normal and parasitized eggs were counted separately under a binocular microscope (×4) to calculate % parasitism.

### 2.2. Female Accessory Gland Substances

The accessory gland of the reproductive organs was dissected out of the female body under low-temperature anesthesia (insects were kept at −20 °C until they stopped moving) and weighed. The gland was immediately broken with fine forceps in an 8 mL glass vial with a polyethylene lid. After removing the gland wall, the liquid substance was preserved at −30 °C prior to the experiments, to examine its ability to protect eggs from desiccation, prevent feeding by ants, and avoid predation by oophagous ladybird beetles. A portion of the substance in the vials was dried for long-term preservation (usually over one month). In this case, a drop of distilled water was added to the dried substance and mixed with fine forceps before the experiments. Therefore, all the experiments did not accurately reproduce the actual substance concentration present in the female glands, but as in the laboratory operation, the substances were dried once secreted to cover the eggs in the field.

**Figure 1:**
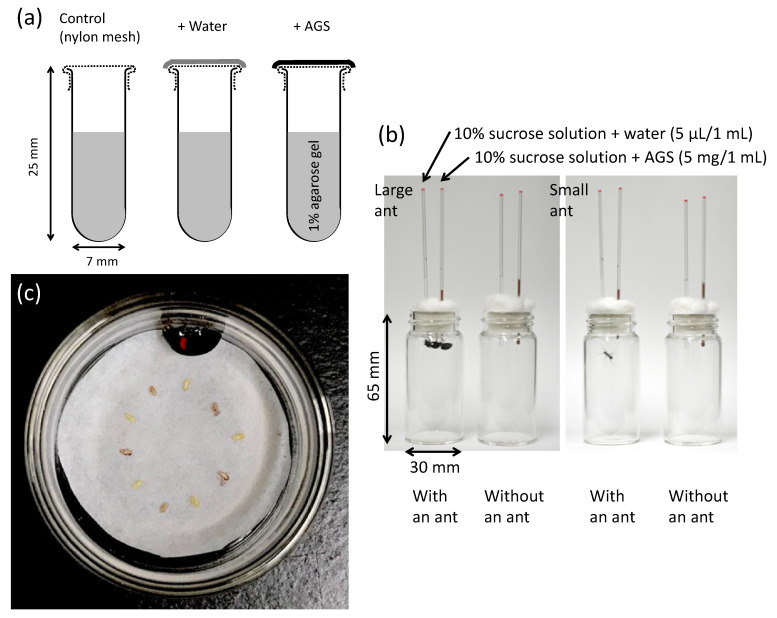
(**a**) The glass tubes containing 1% agarose gel used to examine the effects of female accessory gland substances (AGS) on desiccation rates. The entire opening of the tube was covered with nylon mesh only (control), nylon mesh + water (control for AGS dissolved in distilled water if partly dried), and nylon mesh + AGS. (**b**) Glass vessels used to assess the preference for 10% sucrose solution, with and without AGS in the glass capillaries, of the large and small ant species *Camponotus japonicus* and *Formica japonica*. (**c**) Egg choice of five coated (pale brown) and five uncoated (whitish) eggs with AGS of the egg-eating ladybird beetle *Harmonia axyridis*. The beetle was placed in a Petri dish (26 mm inner diameter) for 24 h.

Desiccation protection was assessed based on the water loss of 1% agarose gel kept at 25 ± 1 °C for 24 h in small glass tubes (7 mm in diameter, 25 mm in height), the openings of which were covered with 0.02 mm nylon mesh (*N* = 3) or nylon mesh + accessory gland substances (*N* = 3) (Figure 1a). The glass tubes covered with nylon mesh + distilled water (*N* = 3) were also used, because a drop of distilled water was added to the partly dried accessory gland substances in the vials for uniform coverage of the nylon mesh with the substances dissolved in water. The experiments were performed once in 10 species and in triplicate (#1–3) in six species. The glass tubes containing the agarose gel were weighed before (W_0_) and after (W_1_) 24 h at 25 °C, and finally after drying in an oven at 80 °C over the next 24 h (W_2_). The water-loss percentage was calculated as (W_0_ − W_1_)/(W_0_ − W_2_) × 100.

Ants are major predators of insects [22] and insect eggs [2] and can usually recognize the presence of repellent compounds. They are ideal for use in bioassays of potential noxious substances [23]. In this study, the predation avoidance function was assessed using the large ant species *Camponotus japonicus* Mayr (Hymenoptera: Formicidae) and small ant species *Formica japonica* Motschoulsky (Hymenoptera: Formicidae) as potential egg predators. These two ant species have highly variable food habits [24]. Workers of these ants were collected from Minamiosawa, Hachioji, Tokyo, central Japan. They were placed in individual glass vials (30 mm in diameter, 65 mm in height) with a piece of wet cotton for water supply and maintained at 25 ± 1 °C (14:10 h L:D cycle). Following a 24 h starvation period, a pair of glass capillaries (1 mm in inner diameter) was placed in each vial through a cotton plug (Figure 1b). These capillaries contained 10% sucrose solution + distilled water (5 µL/mL) and 10% sucrose solution + accessory gland substances (5 mg/mL), respectively. Ten vials with ants and three vials without ants (control) were prepared for each ant species. Before all of the sucrose solution was consumed (after 0.5–6 h for large *C*. *japonicus* and 11–25 h for small *F*. *japonica*), the reduction (L mm) from the initial liquid level was measured using slide calipers. The amount of sucrose solution consumed was calculated as follows: π (0.5 mm)^2^ (L_ant_–L_control_). Following the experiments, the ant head width was measured at the widest part.

Adults of predatory ladybird beetles are also general predators of insect eggs (e.g., [25,26]). In this study, adult ladybird beetles, *Harmonia axyridis* (Pallas) (Coleoptera: Coccinellidae), were collected in Tokyo, Saitama, Yamanashi, and Nagano Prefectures in central Japan. These field-collected individuals were starved for one day and then used for egg feeding experiments. The ovulated mature eggs were dissected out of the female abdomen and placed on a wet filter paper in a small Petri dish (26 mm in inner diameter, 14 mm in height), arranged in an alternating pattern of five uncoated eggs and five coated eggs by dipping them into the thawed female accessory gland substances (Figure 1c). After 24 h, at 25 ± 1 °C (14:10 h L:D cycle), from putting each beetle in the Petri dish, we counted eggs by dividing into three categories, intact, broken, and lost (eaten), as determined under a binocular microscope (×4). If all eggs were intact, the data were excluded from the analysis.

### 2.3. Statistics

Values are shown as mean ± standard error (SE). Pearson’s correlation analysis was used to examine the log-log relationship between the mean female body size (head width) and the mean accessory gland weight among 29 species (Table 1), and the residuals from the estimated regression equation were used to analyze the relative accessory gland size on their phylogenetic relationships of Megaloptera. In the analysis of the ability of accessory gland substances to prevent the desiccation of the eggs, differences in the mean water loss (%) among the three treatment groups were tested by the analysis of variance (ANOVA). The paired *t*-test was used to detect difference in the mean amount of sucrose solution consumed by each ant when the solutions with and without female accessory gland substances were presented simultaneously. In the experiments of the ability of the accessory gland substances to prevent egg predation by the ladybird beetles, differences in frequencies of intact, broken, and lost (eaten) eggs were examined by the chi-square test.

## 3. Results

### 3.1. Egg Masses and Accessory Gland Substances

**Figure 2:**
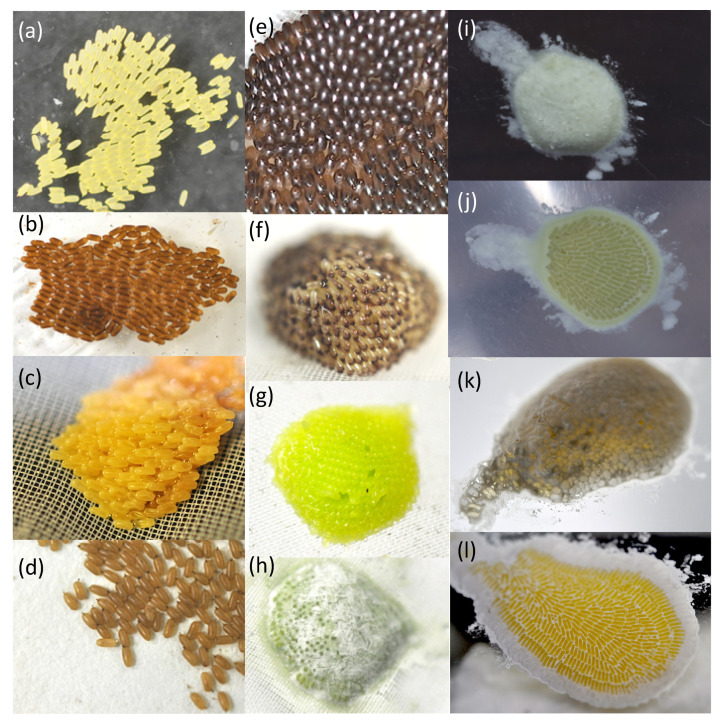
Typical egg masses of Megaloptera. (**a**) *Sialis melania*, in dorsal view; (**b**) *Anachauliodes laboissierei*, in dorsal view; (**c**) *Nigronia serricornis*, in dorsal view; (**d**) *Parachauliodes japonicus*, in dorsal view; (**e**) *Parachauliodes continentalis*, in dorsal view; (**f**) *Protohermes grandis*, in lateral-dorsal view; (**g**) *Protohermes immaculatus*, in dorsal view; (**h**) *Nevromus* sp., in dorsal view; (**i**) *Neoneuromus ignobilis*, in dorsal view; (**j**) ditto, in ventral view; (**k**) *Acanthacorydalis fruhstorferi*, in laterodorsal view; (**l**) ditto, in ventral view.

**Figure 3:**
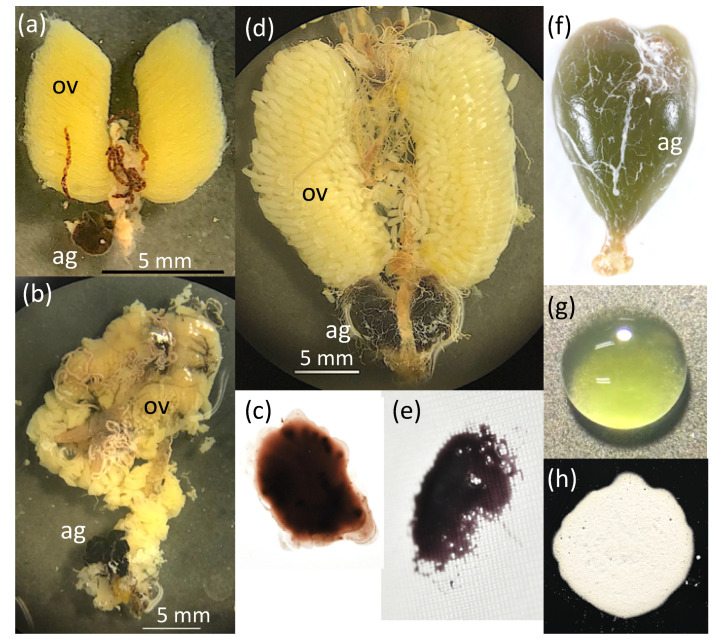
Megaloptera female accessory glands (ag) and ovaries (ov). (**a**) *Sialis melania*, in situ; (**b**,**c**) *Parachauliodes continentalis*, in situ, and accessory gland substance one day after secretion; (**d**,**e**) *Protohermes grandis*, in situ, and accessory gland substance one day after secretion; (**f–h**) *Neoneuromus ignobilis*: (**f**) dissected accessory gland, (**g**) pale green color of the accessory gland substance just after excretion, (**h**) accessory gland substance dried to a hardened white powder.

Female alderflies and fishflies, excluding *Nigronia*, laid single-layered egg masses (Figure 2). In contrast, all species of dobsonflies laid multi-layered egg masses with a hemispherical shape (Figure 2). The female accessory gland was a single pouch in alderflies (Figure 3a), a single elongated tube in fishflies (Figure 3b), and a paired pouch in dobsonflies (Figure 3d,f). Liquid substances present in the female accessory gland were usually pale to dark brown liquid in alderflies and fishflies (Figure 3c). The gland substances were yellow, green, orange, and brown in species of dobsonflies and showed more varied properties: sticky in *Protohermes* species (Figure 3e) and powdered after drying in *Nevromus*, *Neoneuromus*, and *Acanthacorydalis* (Figure 3g,h). In the latter three genera, the egg mass was wholly covered with a hard material (Figure 2).

The female body size of Megaloptera varied greatly in head width and forewing length among genera and species (Table 1). The mean fresh weight of the accessory gland (*y* mg) dissected out of the female abdomen was positively correlated with the mean female head width (*x* mm) in the log-log relationship of 29 species (log_10_ *y* = 4.634 log_10_ *x* − 2.343, *r* = 0.937, df = 27, *p* < 0.001). The mean residual accessory gland size calculated from this equation was larger in *Protohermes* dobsonflies (0.20 ± 0.05, *N* = 13) than *Sialis* alderflies (0.02 ± 0.05 in range, *N* = 2), three genera of fishflies (−0.39, *N* = 1 in *Anachauliodes*; −0.20 ± 0.11 in range, *N* = 2 in *Parachauliodes*; −0.33 ± 0.04, *N* = 4 in *Neochauliodes*), and the other two genera dobsonflies (−0.01 ± 0.08, *N* = 5 in *Neoneuromus*; −0.27 ± 0.06 in range, *N* = 2 in *Acanthacorydalis*).

Two egg masses of *Protohermes grandis* were parasitized by the hymenopteran parasitoid *Oooencyrtus yoshidai* in the field; one in Niigata Prefecture and the other on Sado Island, central Japan; 16.2% and 28.1% of eggs were parasitized, respectively.

### 3.2. Functions of Accessory Gland Substances

**Figure 4:**
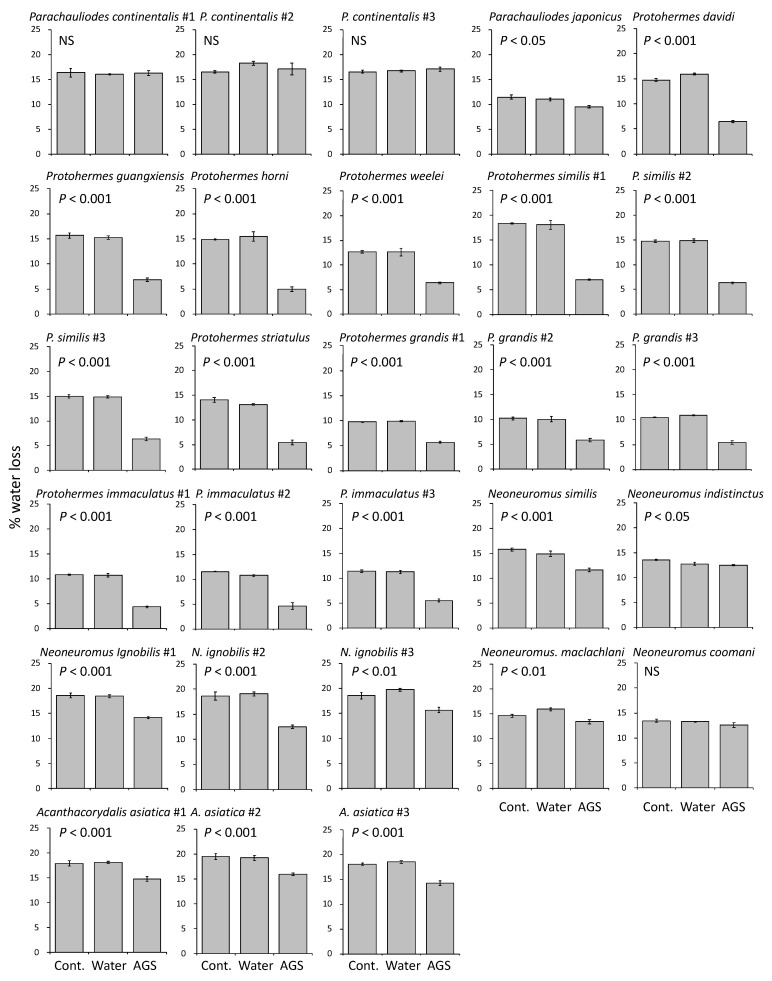
Megaloptera female accessory gland substance effects against dehydration. Mean (± SE, *N* = 3) water loss (%) estimated using agarose gel kept at 25 °C for 24 h (for explanations of Cont., Water, and AGS, see Figure 1a). This experiment was performed in triplicate (#1–3) in *Parachauliodes continentalis*, *Protohermes similis*, *P. grandis*, *P. immaculatus*, *Neoneuromus ignobilis*, and *Acanthacorydalis asiatica*, while there was no replication for other species. The results of ANOVA are also shown (NS: *p* > 0.05).

Female accessory gland substances prevented water loss from the 1% agarose gel, excluding *Parachauliodes continentalis* and *Neoneuromus coomani* (Figure 4). In this experiment, alderflies (*Sialis*) and some fishflies (e.g., *Neochauliodes*) could not be examined because the gland substances sufficient to cover the whole nylon mesh of the tube opening could not be obtained from their small accessory glands.

**Figure 5:**
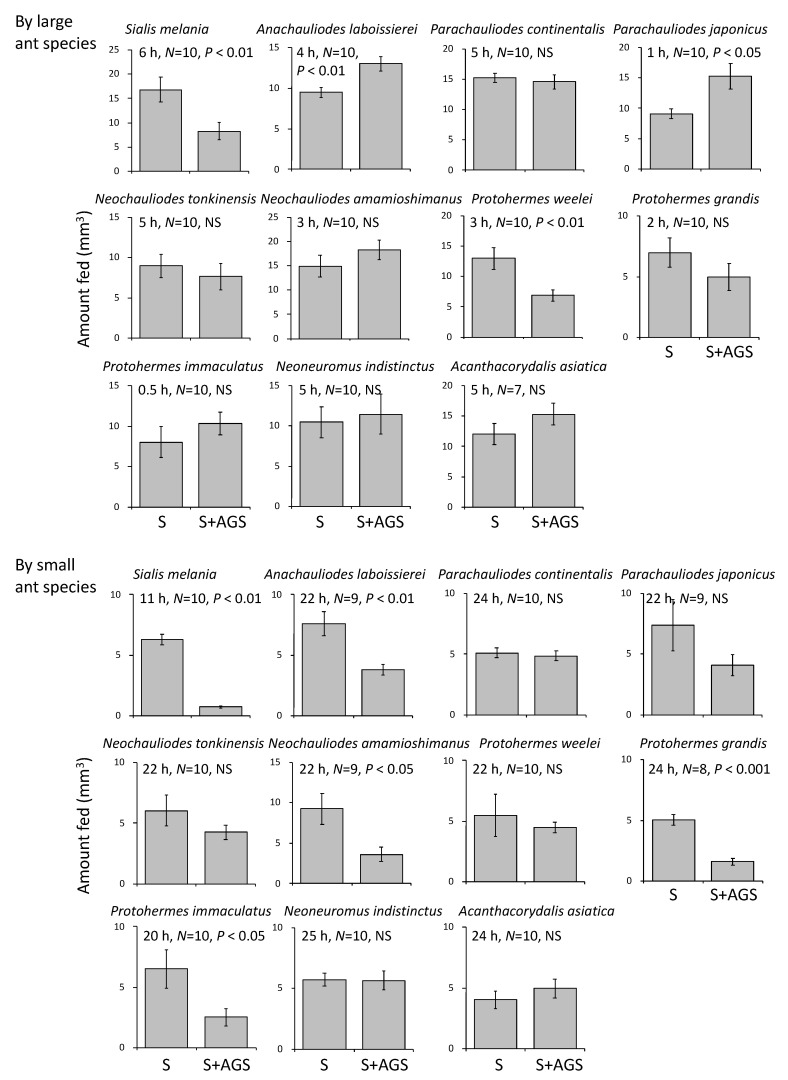
Effect of female accessory gland substances from several species of Megaloptera on avoidance of predation by the large ant species *Camponotus japonicus* (top) and the small ant species *Formica japoninca* (bottom). Two capillaries, one with sucrose solution only (S) and another with sucrose solution plus accessory gland substance (S + AGS), were offered simultaneously to each ant species for 1–25 h (see Figure 1c). *N* is the number of ants examined, and vertical bars show ± SE. The results of paired *t*-tests are also shown (NS: *p* > 0.05).

The head widths of the large and small ant species used in the feeding experiments were 2.24 mm (*N* = 90, SE = 0.06) and 1.16 mm (*N* = 79, SE = 0.01), respectively. The addition of the accessory gland substances to 10% sucrose solution decreased the feeding rate of large ants in two of 11 species tested in the experiment (Figure 5 top). In the small ant species, the rate of feeding on the sucrose solution was decreased by adding the accessory gland substances in five of 11 species tested (Figure 5 bottom).

In the egg choice experiment, the ladybird beetles ate more eggs without accessory gland substances than those with the substances when given the both types of eggs (Table 2). The higher survival of eggs coated with the accessory gland substances was statistically significant in 13 of 15 species, excluding *Sialis tohokuensis* and *P. continentalis*.

## 4. Discussion

### 4.1. Functions of Egg-Coating Substances

**Figure 6:**
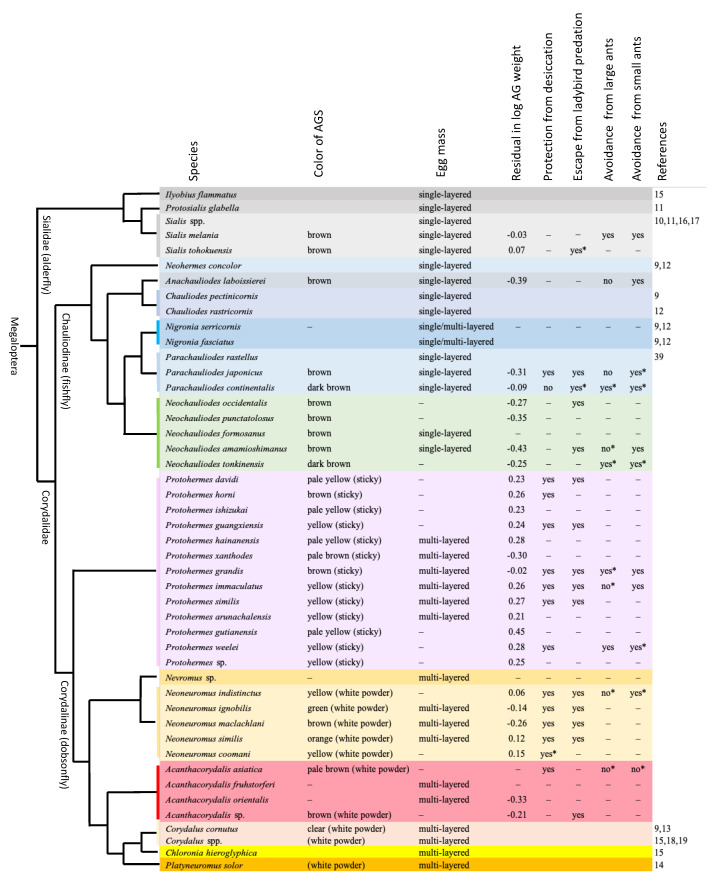
Characteristics of the egg mass and female accessory gland substances (AGS) mapped on the phylogeny of Megaloptera [37]. Different background colors show different genera. * Not statistically significant; – Not examined in this study. For explanation of the log-scale residual of accessory gland (AG) weight, see the statistics section in the main text. With the exception of *Nigronia*, the multi-layered egg masses were all semispherical in shape (see Figure 2).

All species of Megaloptera examined laid their eggs in a mass (Figure 2 and Figure 6). Mature eggs in the ovarioles were milky white, whereas the laid egg masses showed a variety of colors (Figure 2) due to coating with female accessory gland substances of different colors (Figure 3 and Figure 6). Egg or egg mass color may be one of the adapted traits for survival until hatching, particularly for eggs without direct parental care [27]. Crypsis, warning signals, and photoprotection are most important functions of insect egg adaptive coloration [27]. The egg masses of Megaloptera were found on the underside of leaves, tree trunks and branches, and on the surface of rocks near the streams and ponds [9,10,13,16,17]. Yellow and green colors may be used as background-matching camouflage for living leaves, while brown matches tree trunks and branches, and white pale-colored tree trunks or rock surfaces. This type of crypsis may function to prevent predation by diurnal predators visually searching for food, such as birds, lizards, and mammals. The warning coloration of unpalatable or toxic eggs is still unclear in insects [27]. In the present study, we did not examine whether or not the eggs of Megaloptera are unpalatable or toxic for the predators that have the ability to learn by sight. Some pigments have an UV-protecting function, but the egg masses of Megaloptera are usually laid on the underside of the leaves, tree branches, and rocks, because the hatched larvae must fall into the water. This type of egg coloration may be rare in insects [27].

During egg development, the egg coating may help maintain the humidity of eggs. Our assessment of desiccation protection by the accessory gland substances suggested that most species can prevent water loss from the eggs by using these accessory gland substances as coatings (Figure 4). The sticky substances secreted by the female *Protohermes* species seemed to have a strong effect against desiccation. In addition, the hardened white powders produced by *Neoneuromus* and *Acanthacoridalis* were more protective against desiccation compared with the substances produced by *Parachauliodes* (Figure 4). The desiccation tolerance of insect eggs may also arise from physiological processes, such as dormancy and osmoregulation, during embryonic development [28]. However, egg dormancy has not been reported in any species of Megaloptera to date [29]. Egg clustering in a mass is another factor preventing the desiccation of eggs. Single eggs and single-layered egg masses may be disadvantageous with regard to desiccation. In general, egg clustering may reduce the amount of egg surface exposed to ambient conditions, thereby reducing desiccation [30]. In fact, under conditions of low humidity, the hatching rate was lowest in butterfly eggs experimentally arranged in a single loose layer and highest in those arranged in three tightly packed layers [31]. Thus, the multi-layered egg mass in dobsonflies may also promote the retention of humidity.

Most insects coat their eggs with substances produced in the female accessory glands, which is assumed to provide eggs with protection against predators, parasitoids, and microorganisms [2,3,7]. There have been no clear reports of predators eating megalopteran eggs in the field [29]. In our first feeding experiment, two species of ants were used as potential predators, because ants are prominent predators of insect bodies and eggs [2,22]. The accessory gland substances extracted from several species effectively decreased the consumption of sucrose solution by the ants (Figure 5 and Figure 6). This repellent effect against ant feeding may be more advantageous for insects laying eggs in a mass, because all of the eggs in a cluster may be consumed if discovered by foraging worker ants as a result of recruitment [2]. Ladybird beetles are also the general predators of insect eggs (e.g., [25,26]). Our second feeding experiment using adult ladybird beetles showed that the accessory gland substances of most species effectively decreased the risk of predation (Table 2, Figure 6).

**Figure 7:**
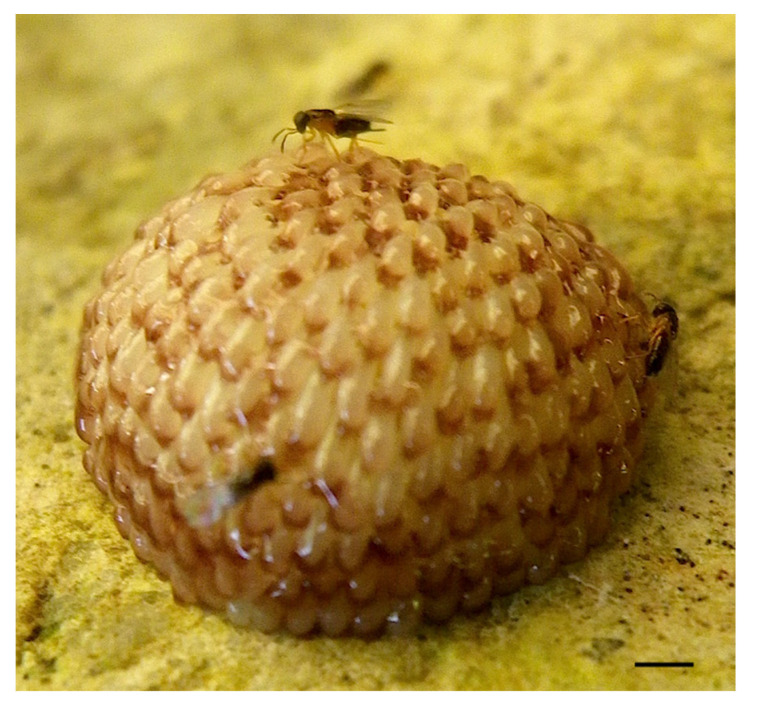
Three females of the egg parasitoid *Ooencyrtus yoshidai* on the multi-layered egg mass with a sticky cover secreted from the female accessory glands of *Protohermes grandis*. Scale bar: 1 mm.

Although five hymenopteran parasitoids (*Trichogramma tajimaense*, *T. semblidis*, *Oooencyrtus longicauda*, *O. protohermesis*, and *O. yoshidai*) and one dipteran parasitoid (*Pseudogaurax idiogenes*) have been reported to parasitize the eggs of Megaloptera (Table 3) [10,18,20,32,33,34,35,36], it is difficult to determine the effectiveness of accessory gland substances for avoidance of egg parasitism. We could not prepare the adult parasitoids and host egg masses simultaneously, but did obtain two egg masses of *P. grandis* parasitized by *O. yoshidai* in the field (Table 3). The parasitism is usually higher in single-layered egg masses of *Sialis* alderflies than multi-layered egg masses of *Protohermes* dobsonflies (with a sticky cover) and *Corydalus* dobsonflies (with a hardened white cover). Interestingly, there have been no reports of parasitism on fishfly egg masses despite being laid in a single layer, as in alderflies. The egg masses are much larger than parasitoid ovipositors (Figure 7), and the eggs located on the surface of the egg mass are expected to be parasitized. However, it is likely that these parasitoids attack the egg masses during oviposition [18,36]. If so, some multi-layered and covered egg masses may be subject to a high degree of parasitism.

The chemical characteristics of the accessory gland substances seem to be species/genera-specific in Megaloptera. However, the chemicals comprising the accessory gland substances are still unknown. In particular, there is interest in the chemical characteristics and synthetic processes of the sticky accessory gland substances secreted by female *Protohermes* and the liquids forming the hardened white covers secreted by female *Neoneuromus*, *Acanthacorydalis*, and their lineages (Figure 3 and Figure 6). Chemical information is required to better understand the structure and function of the diverse female accessory gland substances in Megaloptera. The antimicrobial activity of egg-coating chemicals will be examined in future studies.

### 4.2. Evolutionary Patterns of Egg Mass Characteristics

The evolutionary patterns of the egg mass structure and function in Megaloptera are shown by the molecular phylogenetic tree [37]. The data from the present and previous studies are summarized in Figure 6, although no information on eggs is available for alderflies of *Austrosialis*, *Caribesialis*, *Haplosyalis*, *Indosialis*, *Leptosialis*, and *Stenosialis*; fishflies of *Apochauliodes*, *Archichauliodes*, *Ctenochauliodes*, *Dysmicohermes*, *Madachauliodes, Nothochauliodes*, *Orohermes*, *Platychauliodes*, *Protochauliodes*, *Puri*, and *Taeniochauliodes*; and dobsonflies of *Chloroniella*. Single-layered egg masses may be plesiomorphic because alderflies and fishflies, excluding *Nigronia*, lay eggs in one layer. Egg masses of *Nigronia* are single layered in some masses and multi-layered in others, but not a hemispherical shape, even in the latter cases [9,12]. In contrast, all dobsonflies lay a multi-layered hemispherical egg mass. However, a published photograph of the multi-layered egg mass laid by *Chloronia hieroglyphica* is slightly unclear [15] and should be confirmed in future.

The female accessory gland was a single pouch in alderflies (also see [38]), single elongated tube in fishflies, and paired pouch in dobsonflies. The female accessory gland substances were brown in alderflies and brown to dark brown in fishflies [9,12,39]. In contrast, the accessory gland substances of dobsonflies varied in color among genera and species. The accessory gland weight relative to the body (residual in log scale) differed among genera. *Protohermes* have a larger accessory gland, which may be related to the sticky substances covering the egg mass, while all fishfly genera examined (*Anachauliodes*, *Parachauliodes*, *Neochauliodes*) have a smaller accessory gland. Although based on a small sample size, the accessory gland of *Acanthacorydalis* is relatively small. We must study the reasons why the relative size of female accessory glands varies among genera and species.

## 5. Conclusions

The female accessory gland substances of Megaloptera are used for egg coating and show high diversity in color and other properties. The coloration of egg masses may play a role in crypsis on background substrates such as leaves, tree trunks and branches, and stones. We experimentally demonstrated that the accessory gland substances prevent severe desiccation of eggs during development and avoid predation by oophagous predators such as ladybird beetles and ants. Unfortunately, no information is available on the chemical compounds and synthesis of these egg-coating substances in Megaloptera. Further studies are required for chemical analysis of these substances and to examine other functions such as egg-parasitoid avoidance and antimicrobial activity to understand the evolution of insect egg structure and properties.

## Figures and Tables

**Table 1:** Information on species examined in this study with the mean and standard errors (SEs) of the head width, forewing length, and weight of accessory glands (AGs) in the field-collected females and/or those reared from the field-collected larvae. –: not measured.

Family (Subfamily) Species	Site	Adult State	No. of Females	Head Width (mm)		Forewing Length (mm)		AG Weight (mg)	
				Mean	SE	Mean	SE	Mean	SE
Sialidae									
*Sialis melania* Nakahara	Japan	Field-collected	14	2.74	0.05	15.50	0.19	0.5	0.0
*Sialis tohokuensis* Hayashi & Suda	Japan	Field-collected	4	2.89	0.03	17.15	0.32	0.7	0.1
Corydalidae (Chauliodinae)									
*Anachauliodes laboissierei* (Navás)	China	Field-collected	2	5.73	0.03	51.65	0.45	6.0	0.0
*Nigronia serricornis* (Say)	USA	Reared	1	–		–		–	
*Parachauliodes japonicus* (McLachlan)	Japan	Reared	15	6.25	0.08	44.23	0.66	10.9	1.6
*Parachauliodes continentalis* (Weele)	Japan	Field-collected/Reared	44	6.13	0.04	52.76	0.27	16.4	1.1
*Neochauliodes occidentalis* Weele	China	Reared	9	5.47	0.16	43.16	1.55	6.4	1.5
*Neochauliodes punctatolosus* Liu & Yang	China	Field-collected	2	4.84	0.01	40.47	0.64	3.1	0.5
*Neochauliodes formosanus* (Okamoto)	China	reared	1	–		–		–	
*Neochauliodes amamioshimanus* Liu, Hayashi & Yang	Japan	Reared	13	4.60	0.06	36.05	0.43	2.0	0.4
*Neochauliodes tonkinensis* (Weele)	China	Field-collected	4	5.10	0.09	45.11	1.05	4.8	1.8
Corydalidae (Corydalinae)									
*Protohermes davidi* Weele	China	Reared	5	7.53	0.13	51.49	1.92	89.4	17.5
*Protohermes horni* Navás	China	Reared	2	8.28	0.35	58.56		149.4	36.8
*Protohermes ishizukai* Liu, Hayashi & Yang	China	Field-collected	1	6.02		42.85		31.6	
*Protohermes guangxiensis* Yang & Yang	China	Reared	8	7.26	0.10	50.51	0.67	78.1	5.9
*Protohermes hainanensis* Yang & Yang	China	Field-collected	1	6.59		48.24		54.4	
*Protohermes xanthodes* Navás	China	Field-collected	1	6.00		44.23		9.1	
*Protohermes grandis* (Thunberg)	Japan	Reared/Field-collected	18	6.79	0.09	48.62	0.69	31.3	4.3
*Protohermes immaculatus* Kuwayama	Japan	Reared	27	5.27	0.04	35.79	0.33	18.1	1.6
*Protohermes similis* Yang & Yang	China	Reared	18	6.52	0.06	45.08	0.59	49.6	4.4
*Protohermes arunachalensis* Ghosh	China	Field-collected	1	5.77		47.37		24.8	
*Protohermes gutianensis* Yang & Yang	China	Field-collected	1	5.88		42.53		47.0	
*Protohermes weelei* Navás	China	Reared	4	5.96	0.09	42.14	1.52	34.3	3.5
*Protohermes* sp.	China	Reared	2	5.90	0.60	41.83	4.18	30.5	13.50
*Nevromus* sp.	Vietnam	Reared	1	–		–		–	
*Neoneuromus indistinctus* Liu, Hayashi & Yang	China	Field-collected/Reared	3	8.54	0.16	49.85	0.12	106.9	35.1
*Neoneuromus ignobilis* Navás	China	Field-collected	46	10.07	0.10	60.86	0.51	145.2	17.7
*Neoneuromus maclachlani* (Weele)	China	Reared	3	10.46	0.35	59.10		133.9	25.6
*Neoneuromus similis* Liu, Hayashi & Yang	China	Field-collected/Reared	2	11.14	0.00	67.44	3.04	426.3	221.6
*Neoneuromus coomani* Lestage	China	Reared	1	8.10		54.00		105.0	
*Acanthacorydalis asiatica* (Wood-Mason)	Vietnam	Field-collected	8	–		–		–	
*Acanthacorydalis fruhstorferi* Weele	China	Field-collected	1	–		–		–	
*Acanthacorydalis orientalis* (McLachlan)	China	Field-collected	1	10.49		68.29		114.1	
*Acanthacorydalis* sp.	China	Field-collected	1	12.26		75.55		309.3	

**Table 2:** Predation of eggs of 15 Megaloptera species coated and uncoated with female accessory gland substances (AGS) after 24 h exposure to ladybird beetles, *Harmonia axyridis* (see Figure 1c). *p*-values were determined by the chi-square test.

Family	Subfamily	Species	Egg Treatment	Intact	Broken	Eaten	Total	*p* in *χ*^2^-Tests
Sialidae		*Sialis tohokuensis*	Egsg without AGS	10	8	2	20	0.827
			Eggs with AGS	11	8	1	20	
Corydalidae	Chauliodinae	*Parachauliodes continentalis*	Egsg without AGS	6	7	47	60	0.436
			Eggs with AGS	10	9	41	60	
		*Parachauliodes japonicus*	Eggs without AGS	23	33	39	95	<0.001
			Eggs with AGS	19	59	17	95	
		*Neochauliodes amamioshimanus*	Eggs without AGS	23	1	36	60	0.014
			Eggs with AGS	38	2	20	60	
		*Neochauliodes occidentalis*	Eggs without AGS	25	7	18	50	<0.001
			Eggs with AGS	43	6	1	50	
	Corydalinae	*Protohermes davidi*	Eggs without AGS	17	7	21	45	<0.001
			Eggs with AGS	45	0	0	45	
		*Protohermes guangxiensis*	Eggs without AGS	19	3	33	55	<0.001
			Eggs with AGS	51	4	0	55	
		*Protohermes similis*	Eggs without AGS	7	1	27	35	<0.001
			Eggs with AGS	33	2	0	35	
		*Protohermes grandis*	Eggs without AGS	34	9	62	105	<0.001
			Eggs with AGS	60	19	26	105	
		*Protohermes immaculatus*	Eggs without AGS	35	10	35	80	<0.001
			Eggs with AGS	53	14	13	80	
		*Neoneuromus similis*	Eggs without AGS	8	3	29	40	<0.001
			Eggs with AGS	39	0	1	40	
		*Neoneuromus ignobilis*	Eggs without AGS	17	6	22	45	<0.001
			Eggs with AGS	42	0	3	45	
		*Neoneuromus indistinctus*	Eggs without AGS	3	1	46	50	<0.001
			Eggs with AGS	45	4	1	50	
		*Neoneuromus maclachlani*	Eggs without AGS	8	3	29	40	<0.001
			Eggs with AGS	34	0	6	40	
		*Acanthacorydalis* sp.	Eggs without AGS	5	4	26	35	<0.001
			Eggs with AGS	31	3	1	35	

**Table 3:** Parasitoids known from Megaloptera egg masses: *Trichogramma* (Hymenoptera: Trichogrammatidae), *Ooencyrtus* (Hymenoptera: Encyrtidae) and *Pseudogaurax* (Diptera: Chloropidae). The percentages of parasitized eggs per parasitized egg mass are also shown (N is the number of parasitized egg masses examined).

Host	Parasitoid	Locality	% Eggs Parasitized		Reference
			N	Mean (Range)	
Sialidae (alderfly)					
*Sialis lutaria* (Linnaeus)	*Trichogramma semblidis* (Aurivillius)	Sweden, England			[33]
*Sialis infumata* Newman	*Trichogramma semblidis* (Aurivillius)	North America			[34]
*Sialis californica* Banks	*Trichogramma semblidis* (Aurivillius)	North America	?	(37–65)	[10]
*Sialis rotunda* Banks	*Trichogramma semblidis* (Aurivillius)	North America	?	around 14	[10]
*Sialis cornuta* Ross	*Trichogramma semblidis* (Aurivillius)	North America	12	58.1 ^a^	[20]
*Sialis cornuta* Ross	*Trichogramma semblidis* (Aurivillius)	North America	5	77.7 ^b^	[20]
*Sialis melania* Nakahara	*Trichogramma tajimaense* Yashiro, Hirose & Honda	Japan (Hyogo)	4	56.2 (28.9–94.3)	[32]
Corydalidae: Corydalinae (dobsonfly)					
*Protohermes* sp.	*Ooencyrtus longicauda* Zhang & Zhang	China (Hunan)	1	6.6	[35]
*Protohermes xanthodes* Navás	*Ooencyrtus protohermesis* Zhang & Zhang	China (Beijing)	1	6.8	[35]
*Protohermes grandis* Thunberg	*Ooencyrtus yoshidai* Noyes & Hirose	Japan (Nagano)			[36]
*Protohermes grandis* Thunberg	*Ooencyrtus yoshidai* Noyes & Hirose	Japan (Niigata)	1	16.2	This study
*Protohermes grandis* Thunberg	*Ooencyrtus yoshidai* Noyes & Hirose	Japan (Sado Island)	1	28.1	This study
*Corydalus* spp.	*Pseudogaurax idiogenes* Wheeler ^c^	Brazil (Sao Paulo)		not so high	[18]

^a^ Egg masses laid on natural substrates. ^b^ Egg masses on artificial boards. ^c^ This dipteran parasitid in an egg mass is sometimes parasitized by the unknown small wasp parasitoid.

## Data Availability

The data presented in this study are available on request from the authors.
